# Global knowledge mapping and emerging research trends in the convergence of rheumatoid arthritis and exosomes: A CiteSpace-based visual analysis

**DOI:** 10.1097/MD.0000000000044954

**Published:** 2025-10-10

**Authors:** Zhifeng Zhou, Minghui Hou, Jiayi Ling, Ruilan Liang, Minglin Ou, Min Yang

**Affiliations:** a General Medical Department, The First Affiliated Hospital of Guilin Medical University, Guilin, Guangxi, China; b Laboratory Center, Guangxi Health Commission Key Laboratory of Glucose and Lipid Metabolism Disorders, The Second Affiliated Hospital of Guilin Medical University, Guilin, Guangxi, China; c Laboratory Center, Guangxi Key Laboratory of Metabolic Reprogramming and Intelligent Medical Engineering for Chronic Diseases, The Second Affiliated Hospital of Guilin Medical University, Guilin, Guangxi, China.

**Keywords:** cell-free DNA, CiteSpace, exosomes, fibroblast-like synoviocytes, rheumatoid arthritis

## Abstract

Rheumatoid arthritis (RA) is an autoimmune disorder characterized by persistent synovial inflammation and progressive joint destruction, with a rising global prevalence. By 2050, the age-standardized incidence rate is projected to reach 16.78 per 1,00,000 women and 7.85 per 1,00,000 men. In recent years, exosomes (EXOs) – membrane-bound vesicles (30–150 nm in diameter) that mediate intercellular communication by shuttling proteins, nucleic acids, and lipids – have emerged as promising targets for unraveling RA pathogenesis and developing novel therapies. As key carriers of biological signals, EXOs regulate synovial microenvironment dynamics, including fibroblast-like synoviocyte activation, macrophage polarization, and inflammatory cytokine secretion, thereby playing dual roles in promoting or alleviating RA progression. However, research at this interdisciplinary nexus remains fragmented, lacking systematic synthesis. To address this gap, we employed CiteSpace software to generate a knowledge map from 244 Web of Science Core Collection documents (2000–2025), analyzing global research trends, thematic evolution, and collaborative networks. Key findings include: Growth in academic output: the number of publications continues to rise year by year, with the total number of citations exceeding 9723, significantly enhancing academic influence. Geographical distribution: China leads with 145 publications (centrality = 0.57), followed by the United States. Notably, emerging research hubs in Iran and Germany have recently intensified activity. Thematic priorities: research hotspots cluster around 3 domains: anti-inflammatory activity of EXOs (e.g., M2 macrophage-derived EXOs modulating immune balance), metabolic reprogramming of fibroblast-like synoviocytes regulated by EXO-carried molecules (e.g., circRNAs, miRNAs), and cell-free DNA (cfDNA)-driven immune dysregulation and EXOs-mediated cfDNA clearance. Frontier areas include engineered EXOs delivery systems (e.g., targeted modification for joint enrichment) and cfDNA-mediated immunomodulatory mechanisms. Collaborative landscape: while international partnerships have formed, interdisciplinary integration remains biased toward biomedical sciences. Contributions from materials science and artificial intelligence remain nascent, underscoring opportunities for cross-sector innovation. This analysis provides the first comprehensive knowledge framework for the RA-EXOs interface, offering researchers a roadmap for strategic topic selection, collaboration, and translational innovation toward next generation RA therapies.

## 1. Introduction

Rheumatoid arthritis (RA) is a systemic autoimmune disorder characterized by chronic synovial inflammation, joint destruction, and systemic immune dysregulation.^[1]^ Its rising global prevalence represents a significant public health burden. According to the Global Burden of Disease study, over 17.9 million individuals worldwide lived with RA in 2021, corresponding to an age-standardized prevalence rate of approximately 0.21% (2089 per 1,00,000) and an age-standardized incidence rate of 11.8 per 1,00,000. Incidence is projected to increase further by 2050, reaching 16.78 per 1,00,000 in women and 7.85 per 1,00,000 in men.^[2]^ The epidemiology of RA exhibits substantial spatial and temporal heterogeneity. GBD 2021 data indicate significantly higher ASPR in high-income regions – including North America, Western Europe, and the Caribbean (0.35–0.38%) – compared to low-income regions such as Western Sub-Saharan Africa (0.13%) and South Asia (0.10%).^[3]^ Females exhibit a 2 to 3-fold higher disease risk and earlier average age of onset than males.^[4]^ Notably, RA incidence demonstrates an inverse relationship with socioeconomic status; UK Clinical Practice Research Datalink data reveal a 64% higher incidence in deprived populations relative to the most affluent groups.^[5]^ RA’s global disease burden is substantial, accounting for 30,75,300 disability adjusted life years in 2021^[3]^ – a figure expected to rise with population aging. While biologic agents (e.g., TNF-α inhibitors, IL-6 inhibitors) and targeted synthetic drugs (e.g., JAK inhibitors) have improved patient prognosis, approximately 30% of individuals remain nonresponsive to current therapies.^[6]^ Long-term treatment also carries significant risks of adverse effects. RA pathogenesis involves multifactorial interactions, including genetic susceptibility, environmental triggers (e.g., smoking), dysregulated immune cell activation (such as Th17/Treg imbalance and altered macrophage polarization), and cytokine network dysfunction – particularly the overproduction of IL-6, TNF-α, and IL-1β. Consequently, elucidating the underlying mechanisms of RA pathogenesis and developing targeted diagnostic and therapeutic strategies remain key challenges in current research.

Exosomes (EXOs) have emerged as a significant research frontier in life science and medicine, serving as crucial carriers of intercellular communication. These membrane-bound vesicles, measuring 30 to 150 nm in diameter, facilitate physiological homeostasis regulation and contribute to disease pathogenesis by transporting biomolecules – including proteins, mRNAs, miRNAs, lncRNAs, and DNA – to transmit signals between cells.^[7]^ Their unique biological properties, such as the ability to traverse biological barriers, low inherent immunogenicity, and target modifiability, underpin considerable potential in diverse applications: disease diagnosis (e.g., liquid biopsy), drug delivery (e.g., targeted anti-inflammatory carriers), regenerative medicine (e.g., tissue repair), and immune modulation (e.g., macrophage reprogramming). According to a BCC Research report (May 2024), the global EXOs diagnostics and therapeutics market reached USD 227.5 million in 2023 and is projected to grow to USD 1.3 billion by 2028, reflecting a compound annual growth rate of 42.2%. This rapid development offers novel perspectives for understanding RA mechanisms and developing therapeutic innovations. EXOs are ideal candidates for RA liquid biopsy due to their stable presence in bodily fluids and ease of isolation. Studies indicate significantly altered miRNA profiles in serum-derived EXOs from RA patients, where expression levels of *miR-155* (pro-inflammatory-related), *miR-146a* (NF-κB pathway regulation), and *miR-223* (neutrophil activation) positively correlate with disease activity (DAS28 score).^[8]^ Furthermore, synovial-derived EXOs carrying autoantibodies (e.g., anti-citrullinated protein antibodies) and inflammatory factors (e.g., IL-6, TNF-α) serve as dynamic indicators of disease progression.^[9]^ As reported at the EULAR 2024 conference, single-cell mapping analysis by a Harvard Medical School team revealed significant correlations between synovial tissue EXOs subpopulations and RA disease phenotypes (e.g., CTAPs classification), suggesting potential targets for personalized therapy. EXOs contribute to RA pathology by delivering inflammatory molecules or modulating recipient cell phenotypes. For instance, synovial fibroblast-derived EXOs carrying toll-like receptor 4 (*TLR4*) can promote inflammatory factor secretion in macrophages via NF-κB pathway activation.^[10]^ Additionally, while abnormal cell-free DNA (cfDNA) accumulation exacerbates RA inflammation and macrophage-derived EXOs can degrade cfDNA through DNase I release, DNase activity in EXOs is significantly reduced in RA patients.^[11]^ Engineered EXOs, such as charge-allosteric EXOs (MEX+cP), offer new strategies for synergistic intervention by targeting DNase delivery and inducing macrophage polarization towards the anti-inflammatory M2 phenotype.^[12]^ The natural membrane structure of EXOs renders them ideal drug delivery vehicles. For example, EXOs loaded with anti-inflammatory miRNAs (e.g., *miR-124*) effectively inhibit RA synovial inflammation and exhibit lower immunogenicity than conventional liposomes.^[13]^ Surface modification techniques, including the attachment of targeting peptides or ligands, can further enhance EXOs’ local enrichment within joints. A system developed by researchers at the Institute of Process Engineering, Chinese Academy of Sciences (MEX+cP), significantly inhibited articular cartilage erosion by incorporating a matrix metalloproteinase-sensitive polyethylene glycol (PEG) chain to enable anti-inflammatory factor release within the inflammatory microenvironment. Collectively, these studies lay the groundwork for the clinical translation of EXO-based therapies. While PubMed citations for “RA & EXOs” research grew at an annual rate of 28.6% by 2024, research topics remain highly fragmented, and a systematic interdisciplinary collaborative network has yet to emerge. This fragmentation currently impedes the efficient translation of basic discoveries into clinical practice within this field.

The integration of bibliometrics and knowledge graph technology offers a novel paradigm for analyzing the dynamics of complex disciplines. Tools such as CiteSpace can elucidate the internal logic of disciplinary evolution and potential paradigm shifts through co-citation analysis, keyword burst detection, and clustering visualization.^[14]^ While prior studies have employed these approaches to examine research trends in EXOs within oncology or neurological diseases, a comprehensive analysis of the intersection between RA and EXOs remains unexplored. Three critical issues in this interdisciplinary field warrant further investigation: Temporal and thematic heterogeneity: early research emphasized EXOs’ role in inflammatory delivery (e.g., miRNA-mediated macrophage polarization), whereas recent work has shifted toward engineered EXOs for targeted therapeutics and liquid biopsy applications. Disciplinary imbalance: Biomedical research contributes over 75% of publications, while material sciences and artificial intelligence driven studies on novel drug delivery systems remain underdeveloped. Geographic fragmentation: China and the United States account for 70% of highly cited research, yet cross-regional collaboration remains limited. To address these gaps, this study employs CiteSpace to conduct a systematic analysis of 244 publications on RA-EXO research from Web of Science Core Collection (WoSCC; 2000–2025). We aim to: identify high frequency keywords and core research domains; map the evolution of research hotspots and emerging frontiers; Analyze international collaboration networks and high-impact institutions; and propose translational pathways and future research priorities. This work provides researchers with a panoramic overview of the field, facilitating strategic topic selection and collaboration development. Concurrently, by identifying emergent research directions, it establishes a theoretical foundation for innovating therapeutic interventions.

## 2. Materials and methods

### 2.1. Data sources

**Figure 1.**
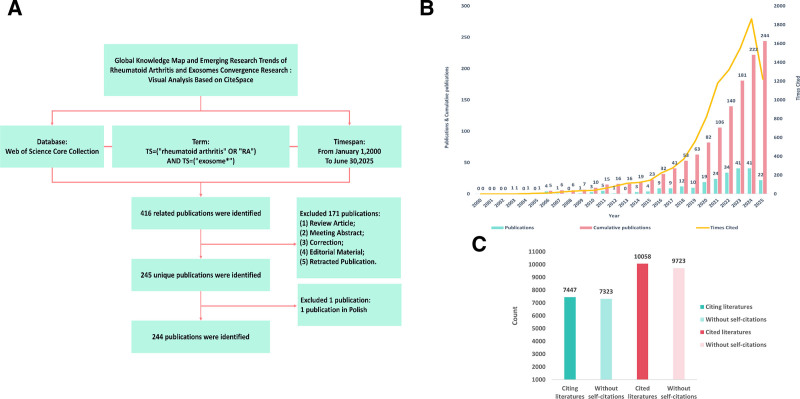
(A) Flowchart of the literature screening process and bibliometric analysis steps in this study. (B) Annual number and citation frequency of papers on rheumatoid arthritis and exosomes during 2000 to 2025. (C) Visual presentation of cited and referenced literature.

WoSCC, a premier academic search platform under Clarivate Analytics, derives its core value from constructing a global academic research network. This database enables in-depth mining of literature associations through a multidimensional indexing system, distinguished by 3 key features: A rigorous journal selection mechanism – considering impact factor (IF) percentile, geographic balance, and disciplinary representativeness – has yielded a refined citation dataset spanning 178 disciplinary fields; Comprehensive inclusion of citation genealogy data (from 1900 to present), supporting both forward and backward citation tracking; and structured data features particularly conducive to bibliometric visualization analysis. These features include a full-field metadata export function (encompassing author institutions, funding, keyword co-occurrence, etc), providing essential elements for knowledge graph construction; an embedded Essential Science Indicators (ESI) hot paper threshold algorithm for automatic identification of emergent literature; and API integration with analytical tools (e.g., CiteSpace, VOSviewer) enabling direct transformation of raw data into bibliographic databases and network topology diagrams. For instance, co-cited author networks can be generated using the “cited references” field, while research trend timelines can be plotted using the “publication year” field – a multi-granular data structure unattainable in standard databases. Studies indicate that approximately 92% of SCI/SSCI journal evaluation research utilizes WoSCC, with its standardized citation data providing a reliable foundation for visual analysis. For this study, data were extracted from WoSCC on July 10, 2025, using the search query: TS=(“rheumatoid arthritis” OR “RA”) AND TS=(“exosome*”). The search was refined by: Document Type: Article; Language: English; and Publication Date: January 1, 2000 to June 30, 2025. System default parameters were applied, while Review Articles, Meeting Abstracts, Corrections, Editorial Material, and Retracted Publications were excluded to ensure analytical accuracy and objectivity. This yielded 244 relevant publications, exported in the “download_XX.txt” plain text format for subsequent analysis and visualization in CiteSpace. The literature screening process is detailed in Figure 1A.

### 2.2. Research methods

This study employed the CiteSpace scientometric analysis tool to systematically map knowledge domains within the target literature. The analysis parameters were set to a time span of 2000–2025 with a 1-year time slice interval. Multiple node types were comprehensively examined, including geographic distribution, academic institutions, individual researchers, co-citation networks, and subject terms. During data processing, the top 50% most cited publications were selected, and the Pathfinder algorithm was applied to prune and optimize the network structure. The investigation commences with a bibliometric assessment of publication citation characteristics to evaluate the evolutionary trajectory of academic influence within the field. Subsequently, a multilevel research collaboration network – encompassing international, interinstitutional, and inter-scholar relationships – was constructed to identify core research groups and potential collaborative innovation opportunities. In the network visualizations, circular markers represent distinct research entities, with their diameters proportional to academic output volume. Connecting lines characterize collaborative relationships, their widths intuitively reflecting the frequency and intensity of collaboration. To probe research content, co-citation analysis was implemented to identify seminal works and trace their intellectual legacy. Notably, the study integrates thematic cluster detection to identify key research foci across distinct periods and burst detection to effectively capture the dynamic evolution of research hotspots. Collectively, this multifaceted analysis systematically delineates the research landscape at the intersection of RA and EXOs over the past 25 years, providing subsequent researchers with valuable theoretical foundations and methodological insights.

### 2.3. Ethical approval and patient consent

As this study is a bibliometric analysis utilizing publicly available literature data from the WoSCC database, it did not involve any direct human or animal subjects, collection of primary patient data, or clinical interventions. Therefore, ethical committee/institutional review board approval and informed consent from patients were not required for this research.

## 3. Results

### 3.1. Analysis of annual output trends and citation trends

This study employed annual statistical analysis to chart research trends in the field of RA and EXOs from 2000 to 2025 (Fig. 1B). The results demonstrate a consistent increase in publications over this 25-year period, reflecting substantial academic interest within rheumatology. The inaugural paper appeared in 2003, with annual output peaking at 41 publications in 2023. By 2025, cumulative publications in this field exceeded 200. Citation metrics revealed even more pronounced growth, particularly between 2021 and 2025, when annual citations increased by over 1000. Figure 1C distinguishes citations received from references cited. After excluding self-citations, the total number of citations was 7323; including self-citations, this figure reached 9723, yielding an average of 39.85 citations per paper. These metrics underscore the enduring academic influence and significant scientific value of research in this domain.

### 3.2. Country/region visualization analysis

**Figure 2.**
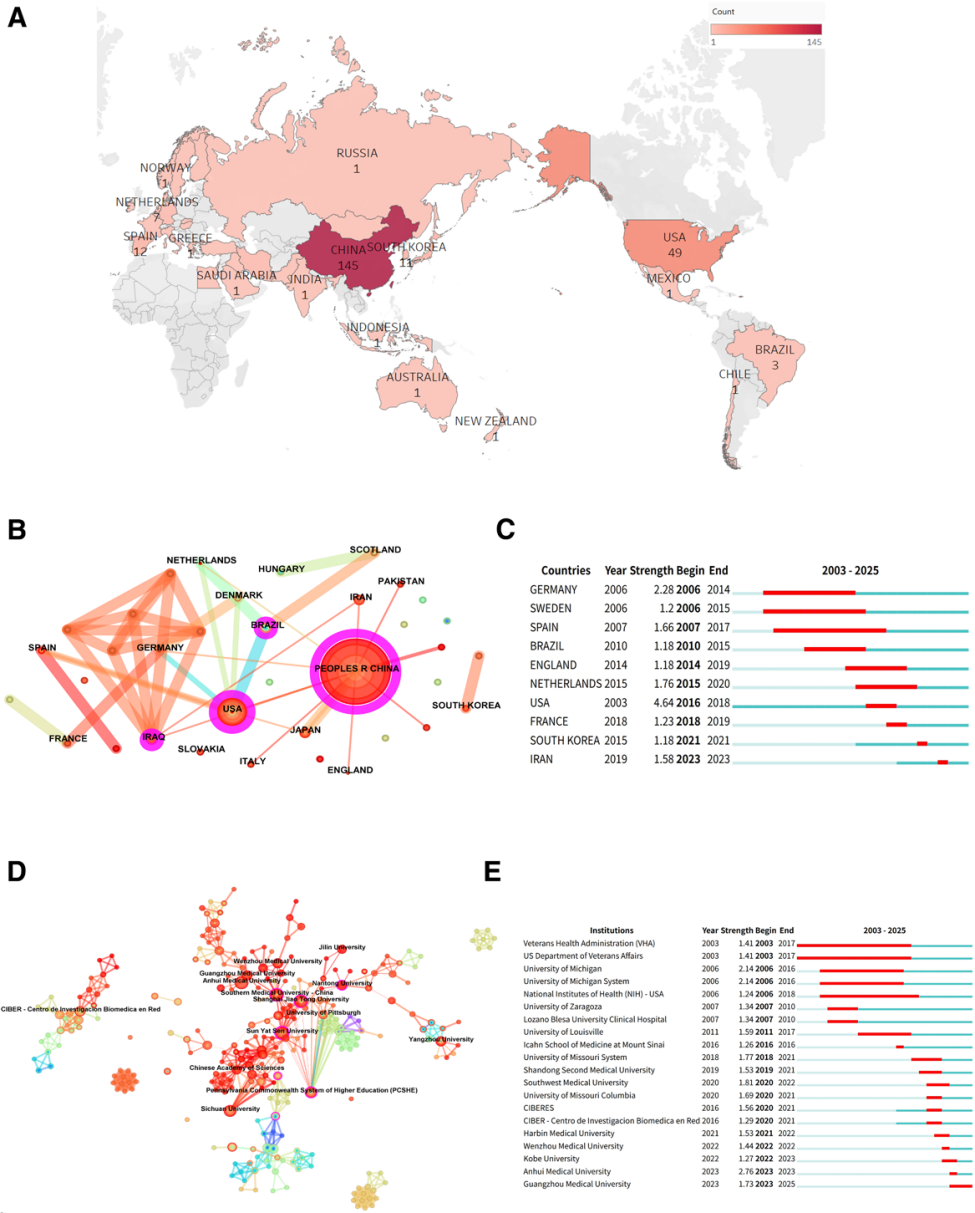
(A) Global distribution of countries/regions involved in rheumatoid arthritis and exosomes research. (B) Visualization map of countries/regions. Each node represents a country or region, and the node size is related to the number of published articles. Lines connecting the nodes represent collaborations between countries/regions. (C) The top 10 countries/regions with the most significant sudden increase in the number of articles published. Blue bars represent the timeline and red bars represent the start and end years of the burst duration. (D) Institution visualization map. Each node represents an institution, scaled according to the number of published articles. Lines connecting the nodes represent interinstitutional collaborations. (E) Top 20 institutions with the most significant sudden increase in the number of published articles.

We generated a global distribution map depicting regional publication output in this field (Fig. 2A), visually representing the relative activity levels and scholarly contributions of different countries/regions. Econometric analysis using CiteSpace’s country/region module (Fig. 2B) reveals a research network comprising 37 participating entities and 44 collaborative links, with a network density of 0.0661. This indicates the emergence of an incipient international cooperative framework within the field. The academic standing of each nation was evaluated using centrality metrics, which quantify a node’s role as a hub within the research network; higher values denote greater academic influence. Table 1 lists the top 10 countries/regions ranked by publication volume and centrality. China leads in both publication volume (145 articles) and centrality (0.57). This prominent productivity and influence arise from several interconnected factors: Policy guidance and substantial resource investment have served as key drivers. Major national initiatives, such as the National Natural Science Foundation of China, have explicitly prioritized RA-EXOs research within their core funding strategies. Illustrative examples include the charge-regulated EXOs research conducted by the Institute of Process Engineering, Chinese Academy of Sciences, which has received sustained national program support and advanced to the preclinical stage. Furthermore, China’s large RA patient population (>5 million cases) and demographic aging trends have concentrated research resources in this area. China has also systematically established a standardized EXOs research infrastructure encompassing isolation and purification techniques, characterization databases, and experimental protocols, effectively lowering technical barriers to entry. Collectively, these factors have positioned China as a leader in this domain. The United States and Spain rank within the top 3 countries by publication volume and represent other significant contributors. Burst detection analysis (Fig. 2C) indicates a notable surge in publication output and scholarly interest from South Korea and Iran over the past 2 years. This trend signals a rapid restructuring of the international research landscape, reflecting the rise of emerging research hubs and underscoring the growing global attention focused on this interdisciplinary field.

**Table 1:** Top 10 countries/regions with the publications and centrality.

Rank	Country/region	Count	Country/region	Centrality
1	China	145	China	0.57
2	USA	49	Iraq	0.29
3	Spain	12	USA	0.27
4	Japan	11	Brazil	0.13
5	South Korea	11	Germany	0.09
6	Germany	7	Spain	0.07
7	Iran	7	Scotland	0.07
8	Netherlands	7	Netherlands	0.06
9	England	5	Japan	0.00
10	Brazil	3	South Korea	0.00

### 3.3. Visualization and analysis of research institutions

The CiteSpace institutional collaboration network analysis (Fig. 2D) identified 391 research institutions engaged in this field globally, forming a sparse collaboration network with a density of 0.0122. Academic contribution analysis (Table 2) revealed a pronounced dominance of Chinese institutions: 90% of the top 10 institutions by publication volume and 80% of the top 10 by network centrality are based in China. Specifically, Anhui Medical University (n = 6, 5.56%), Sun Yat-sen University (n = 5, 4.63%), and Sichuan University (n = 4, 3.70%) ranked highest in publication output. The Anhui Medical University team analyzed RNA sequencing data from serum exosomal lncRNAs and fibroblast-like synoviocytes (FLS), identifying differentially expressed lncRNAs and mRNAs to construct a competing endogenous RNA (ceRNA) network. They proposed 4 RA-associated serum exosomal lncRNAs (*DLEU2, FAM13A-AS1, MEG3*, and *SNHG15*) as biomarkers for intercellular communication. Their findings suggest these ceRNA networks may regulate FLS-characterizing genes, contributing to malignant phenotypes and adaptive synovial microenvironments in RA.^[15]^ Sun Yat-sen University emerged as a key academic hub (centrality = 0.09), reflecting its disciplinary leadership. Its research team demonstrated that EXOs derived from gingival mesenchymal stem cells significantly attenuated arthritis symptoms and bone erosion in collagen-induced arthritis (CIA) mice. This effect – mediated by modulation of Th17/Treg immune homeostasis and suppression of the IL-17RA-Act1-TRAF6-NF-κB signaling pathway – exceeded that of the parent stem cells, suggesting a promising cell-free therapeutic strategy for RA.^[16]^ Research activity trend analysis (Fig. 2E) indicates early leadership by institutions including the Veterans Health Administration (VHA), the US Department of Veterans Affairs, and the University of Michigan. In contrast, Kobe University, Anhui Medical University, and Guangzhou Medical University have substantially increased their academic influence in recent years.

**Table 2:** Top 10 Institutions with the publications and centrality.

Rank	Institution	Count	Institution	Centrality
1	Anhui Medical University	6	Sun Yat-Sen University	0.09
2	Sun Yat-Sen University	5	Southern Medical University	0.09
3	Sichuan University	4	Jinan University	0.08
4	Southwest Medical University	4	Guangzhou University of Chinese Medicine	0.06
5	Yangzhou University	4	China Academy of Chinese Medical Sciences	0.03
6	Pennsylvania Commonwealth System of Higher Education (PCSHE)	3	Tabriz University of Medical Science	0.03
7	Chinese Academy of Sciences	3	Peking Union Medical College	0.03
8	China Medical University	3	Pennsylvania Commonwealth System of Higher Education (PCSHE)	0.02
9	China Academy of Chinese Medical Sciences	3	Chinese Academy of Sciences	0.01
10	Southern Medical University	3	Sichuan University	0.01

PCSHE = Pennsylvania Commonwealth System of Higher Education.

### 3.4. Visualization and analysis of authors’ collaboration networks

**Figure 3.**
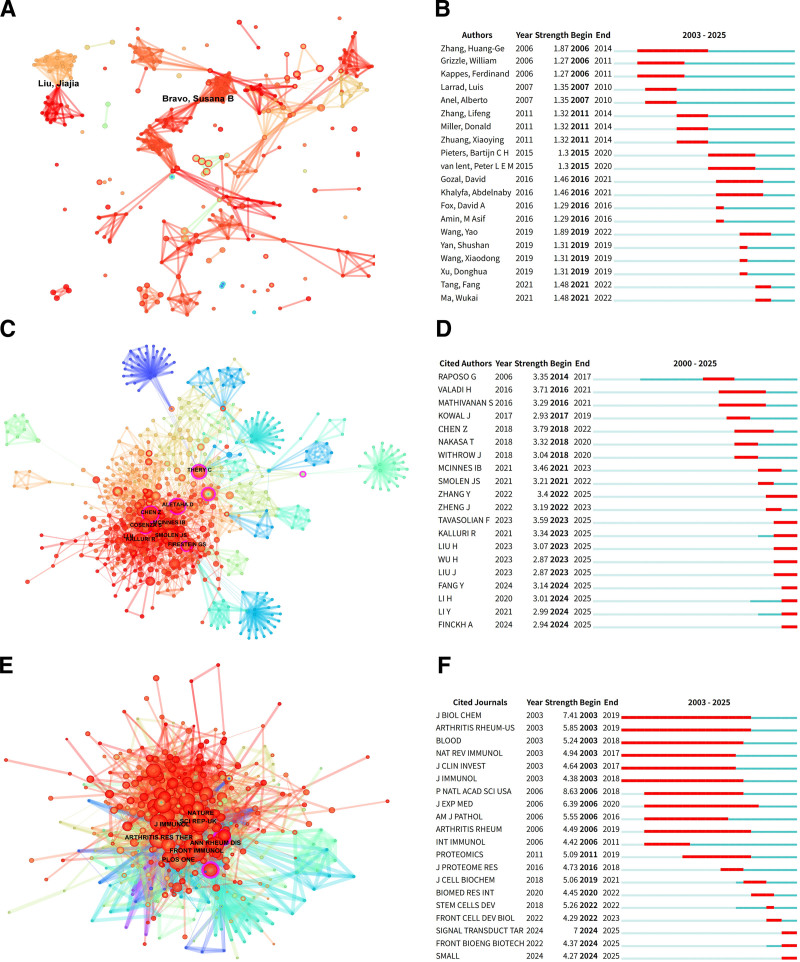
(A) Author visualization map. Each node represents an author and the node size reflects the number of published articles. Lines between nodes represent collaboration between authors. (B) Top 20 authors with the most significant sudden increase in the number of publications. (C) Author co-citation. Node size is proportional to the number of citations. (D) The 20 authors with the sudden increase in citations. (E) Journal co-citation. Node size corresponds to number of citations. (F) Top 20 journals with the strongest citation bursts.

Visualization and analysis of author collaboration networks yield significant insights into research collaboration dynamics, encompassing scholars’ academic productivity, influence, research trajectories, and collaborative patterns. In this study, author data were extracted and analyzed using the Author function module of CiteSpace software. Figure 3A illustrates the overall collaboration network within the field, comprising 1886 researcher nodes connected by 5966 collaborative links, with a network density of 0.0034. To systematically evaluate academic contributions, researchers were ranked by the average citation frequency of their publications (Table 3). The highest-ranked researcher, Yao Wang, developed folic acid-PEG-cholesterol-modified EXO-based biomimetic nanoparticles for efficient delivery of dexamethasone sodium phosphate. This approach enhanced therapeutic efficacy in RA, reduced bone erosion, and diminished systemic toxicity.^[17]^ The second-ranked researcher, Dan Liu, identified *miR-106b* in synovial fibroblast-derived EXOs as a promoter of RA progression. This microRNA inhibits chondrocyte proliferation and migration, accelerates apoptosis, and disrupts the *RANKL*/*RANK*/*OPG* system by targeting pyruvate dehydrogenase kinase 4 (*PDK4*).^[18]^ Suppression of *miR-106b* alleviated RA symptoms, suggesting a novel therapeutic target. Yuxuan Fang, ranked third, revealed that the exosomal long noncoding RNA *NEAT1* (nuclear paraspeckle assembly transcript 1), derived from peripheral blood mononuclear cells, promotes RA development via the *miR-23a*/*MDM2*/*SIRT6* Axis.^[19]^ *NEAT1* downregulates *miR-23a* expression, thereby activating the *MDM2*/*SIRT6* axis to stimulate RA cell proliferation, inflammatory factor secretion, and NF-κB-mediated inflammatory responses. Burst detection analysis (Fig. 3B) indicated that HuangGe Zhang, William Grizzle, and Ferdinand Kappes were among the earliest contributors to the field, whereas Donghua Xu, Fang Tang, and Wukai Ma represent recent entrants.

**Table 3:** Top 10 Most influential authors.

Rank	Author	Count	Total of times cited	Average of times cited	*H*-index	Centrality
1	Yao Wang	3	313	104.33	3	0.00
2	Dan Liu	3	90	30.00	3	0.00
3	Yuxuan Fang	3	90	30.00	3	0.00
4	David Gozal	3	90	30.00	3	0.00
5	Abdelnaby Khalyfa	3	86	28.67	3	0.00
6	Jie Tian	3	52	17.33	2	0.00
7	Marije I Koenders	3	42	14.00	3	0.00
8	Onno J Arntz	3	42	14.00	3	0.00
9	Zhuyi Ji	3	42	14.00	3	0.00
10	Yukai Huang	3	42	14.00	3	0.00

### 3.5. Visualization and analysis of co-cited author networks

The author co-citation network identifies scholars of significant academic influence in RA and EXO research. This network establishes connections by quantifying the frequency with which pairs of scholars are simultaneously cited within the same publications. Following analysis using a path-finding algorithm, the final network comprises 1952 nodes and 6863 links. Researchers receiving more than 10 citations were annotated with first-author information. Through visual analysis (Fig. 3C) and quantitative assessment (Table 4), we identified prominent scholars exhibiting both high citation counts and strong network centrality metrics. For instance, Stella Cosenza (n = 21 citations, centrality = 0.08) ranked among the top 10 scholars for both indicators. Such individuals, demonstrating high academic citation impact and network centrality, can be recognized as authoritative experts in this field. Cosenza’s research compared mesenchymal stem cell-derived EXOs with microparticles, revealing that EXOs exert superior anti-inflammatory effects in inflammatory arthritis models.^[20]^ In vitro experiments demonstrated that both inhibited T- and B-lymphocyte proliferation and induced regulatory T-cell (Treg) differentiation; however, EXOs significantly increased the Treg ratio. In a CIA model, EXOs markedly reduced joint swelling and bone destruction, promoted regulatory B-cell production, and diminished plasma cell differentiation. While cryopreservation compromises EXO structure and abolishes their activity, freshly prepared EXOs exhibit enhanced efficacy by modulating inflammatory factors such as IL-10 and IL-6. These findings provide a key rationale for extracellular vesicle therapy in arthritis. Furthermore, influential scholars can be identified through citation burst analysis, which detects sharp increases in citation frequency over short periods. Figure 3D reveals Zhe Chen as having the highest burst strength (Strength = 3.79). Chen’s work demonstrated that exosomal delivery of mesenchymal stem cell-derived *miR-150-5p* targets and downregulates *MMP14* and vascular endothelial growth factor (*VEGF*) expression. This mechanism significantly alleviated joint destruction, reduced inflammation, and inhibited synovial cell migration, invasion, and angiogenesis in a CIA mouse model.^[21]^

**Table 4:** Top 10 co-cited authors.

Rank	Co-cited author	Co-citations	Co-cited author	Centrality
1	Josef S Smolen	36	Stefano Alivernini	0.16
2	Zhe Chen	33	Nunzio Bottini	0.14
3	Iain B McInnes	26	Chi-Hsien Liu	0.12
4	Daniel Aletaha	23	Jose M Álvaro-Gracia	0.12
5	Raghu Kalluri	21	Peter J Barnes	0.11
6	Stella Cosenza	21	F C Arnett	0.10
7	Gary S Firestein	16	Yili Wang	0.09
8	Fataneh Tavasolian	14	Mohammed Alghamdi	0.09
9	Lu Zhang	14	Mojtaba Abbasi	0.09
10	David D Brand	12	Stella Cosenza	0.08

### 3.6. Analysis of highly produced journals

Bibliometric analysis identified a total of 244 publications in the field of RA and EXOs research, distributed across 75 academic journals. The distribution of the top 10 journals is presented in Table 5. Using 2025 Journal Citation Reports data, the latest IF for each journal was retrieved as a key indicator of academic influence. Results indicate that these 10 journals had an average IF of 5.95 and collectively published 78 articles, representing 31.97% of the total publications. Regarding article volume, *International Immunopharmacology* ranked first with 22 papers (9.02%), followed by *Frontiers in Immunology* (5.74%), *Arthritis Research & Therapy* (4.10%), *Cells* (2.87%), and *Heliyon* (2.87%). Although *Journal of Nanobiotechnology* ranked sixth in publication count (5 articles), it achieved the highest IF among the cohort, reflecting its prominence in this field and the well-recognized quality of its publications within the academic community.

**Table 5:** Top 10 published journals.

Rank	Journal	Output	Percentage	IF	JCR	Publisher
1	International Immunopharmacology	22	9.02	4.7	Q1	Elsevier
2	Frontiers in Immunology	14	5.74	5.9	Q1	Frontiers Media S.A.
3	Arthritis Research & Therapy	10	4.10	4.6	Q1	BioMed Central
4	Cells	7	2.87	5.2	Q2	MDPI (Basel, Switzerland)
5	Heliyon	7	2.87	3.6	Q1	Elsevier
6	Journal of Nanobiotechnology	5	2.05	12.6	Q1	BioMed Central
7	Journal of Orthopaedic Surgery and Research	4	1.64	2.8	Q1	BioMed Central
8	Materials & Design	3	1.23	7.9	Q1	Elsevier BV
9	PLoS One	3	1.23	2.6	Q1	Public Library of Science
10	Acta Biomaterialia	3	1.23	9.6	Q1	Elsevier BV

IF = impact factor, JCR = Journal Citation Reports.

### 3.7. Visual analysis of co-cited journals

Based on a visual analysis of the journal co-citation network, this study methodically delineates the core scholarly communication channels within the RA and EXOs research domain. By constructing a journal association map (Fig. 3E), we identified key journal clusters that serve dual functions as repositories of established knowledge and hubs for academic dissemination. As detailed in Table 6, journals including *Annals of the Rheumatic Diseases* (n = 58, centrality = 0.09), *Scientific Reports* (n = 52, centrality = 0.09), *Nature Reviews Rheumatology* (n = 49, centrality = 0.09), and *Theranostics* (n = 44, centrality = 0.12) ranked among the top 10 based on co-citation frequency and network centrality. Notably, these journals maintained average annual citation rates exceeding 40, underscoring their pivotal role in facilitating interdisciplinary knowledge integration. Furthermore, burst detection analysis revealed significant citation bursts across 20 journals (Fig. 3F). Proceedings of the National Academy of Sciences (PNAS) exhibited the highest burst intensity (8.63), active from 2006 to 2018, signifying the transformative impact of its published findings within this field during that period. Collectively, these analyses indicate that journals demonstrating high co-citation frequency coupled with strong network centrality typically fulfill dual roles as central knowledge repositories and platforms for disseminating cutting edge research.

**Table 6:** Top 10 co-cited journals.

Rank	Co-cited journal	Co-citations	IF	JCR	Co-cited journal	Centrality	IF	JCR
1	Frontiers in Immunology	61	5.9	Q1	Stem Cell Research & Therapy	0.15	7.3	Q1
2	Annals of the Rheumatic Diseases	58	20.6	Q1	Theranostics	0.12	13.3	Q1
3	Scientific Reports	52	3.9	Q1	Nature Communications	0.10	15.7	Q1
4	Journal of Immunology	50	3.4	Q2	Journal of Cellular Physiology	0.10	4.0	Q1
5	Nature Reviews Rheumatology	49	32.7	Q1	Annals of the Rheumatic Diseases	0.09	20.6	Q1
6	Arthritis Research & Therapy	48	4.6	Q1	Scientific Reports	0.09	3.9	Q1
7	Autoimmunity Reviews	45	8.3	Q1	Nature Reviews Rheumatology	0.09	32.7	Q1
8	International Immunopharmacology	45	4.7	Q1	Nature	0.09	48.5	Q1
9	Theranostics	44	13.3	Q1	Rheumatology	0.09	4.4	Q1
10	Arthritis & Rheumatology	44	10.9	Q1	Clinical and Experimental Rheumatology	0.08	3.3	Q2

IF = impact factor, JCR = Journal Citation Reports.

### 3.8. Analysis of highly cited articles

**Figure 4.**
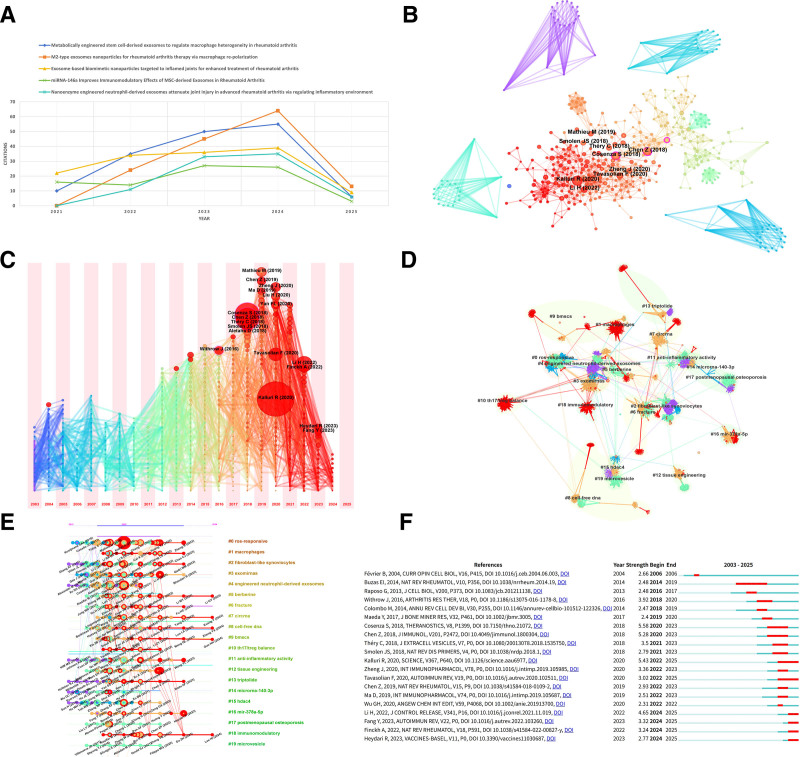
(A) Annual citation distribution of the 5 most cited articles. (B) Total cited references co-occur. This figure shows the names of references that were cited more than 5 times in total. Node size is proportional to frequency of occurrence. (C) Co-cited reference time zone mapping. Node size is proportional to count value. Co-cited references that first appeared in the same year were aggregated to the same time zone using a time slice of “1” year, incrementing from left to right. (D) Clustering of co-cited references. Different colors represent different clusters. Each node represents a co-cited reference, and the number on the node indicates the cluster to which the reference belongs. The smaller the number of labels, the more references in that cluster. (E) Timeline of co-cited reference clustering. For each cluster, the position of each node represents when the literature was published and the node size represents the number of citations. (F) Top 20 references with the strongest citation bursts.

Table 7 presents the 10 most cited papers in RA and EXO research over the past 5 years. Reviewing these articles offers significant insight into current research trends and future directions within this field. Figure 4A illustrates the citation distribution of the top 5 highly cited publications. The foremost cited article, authored by Dong Gil You (Sungkyunkwan University), peaked at 55 citations in 2024. You et al developed engineered EXOs (DS-EXOs) targeting macrophages by modifying the surface of adipose-derived stem cells via metabolic glycan engineering and click chemistry.^[22]^ These DS-EXOs utilize surface-conjugated dextran sulfate to bind macrophage scavenger receptor A (SR-A) in inflamed joints, significantly enhancing their enrichment at articular sites. The study confirmed that DS-EXOs effectively promote polarization of pro-inflammatory M1 macrophages toward the anti-inflammatory M2 phenotype through delivery of key miRNAs (*let-7b-5p* and *miR-24-3p*). Notably, DS-EXOs achieved therapeutic efficacy equivalent to conventional EXOs at one-tenth the dose. In a CIA murine model, systemic administration of DS-EXOs markedly reduced joint inflammation and inhibited bone erosion, demonstrating a novel cell-free therapeutic strategy for RA. The second most cited article, by Hui Li (Southwest Medical University), reached 64 citations in 2024. Li et al designed a nanoparticle delivery system utilizing M2 macrophage-derived EXOs to co-deliver anti-inflammatory interleukin-10 (IL-10) plasmid DNA and betamethasone sodium phosphate.^[23]^ This system significantly suppressed pro-inflammatory cytokines (e.g., TNF-α, IL-1β) while elevating IL-10 levels by promoting M1-to-M2 macrophage polarization. In an RA murine model, it exhibited precise inflammation targeting, potent anti-inflammatory activity, significant joint protection, and minimal toxicity, establishing a novel biocompatible treatment approach. The average citation count for the top 5 articles exceeded 100, underscoring the sustained academic impact of their research designs and findings.

**Table 7:** Top 10 cited articles.

Rank	Title	First author	Journal	Year published	Total citations
1	Metabolically engineered stem cell-derived exosomes to regulate macrophage heterogeneity in rheumatoid arthritis	Dong Gil You	Science Advances	2021	156
2	M2-type exosomes nanoparticles for rheumatoid arthritis therapy via macrophage repolarization	Hui Li	Journal of Controlled Release	2022	146
3	Exosome-based biomimetic nanoparticles targeted to inflamed joints for enhanced treatment of rheumatoid arthritis	Feili Yan	Journal of Nanobiotechnology	2020	140
4	miRNA-146a Improves Immunomodulatory effects of MSC-derived exosomes in rheumatoid arthritis	Fataneh Tavasolian	Current Gene Therapy	2020	86
5	Nanoenzyme engineered neutrophil-derived exosomes attenuate joint injury in advanced rheumatoid arthritis via regulating inflammatory environment	Lei Zhang	Bioactive Materials	2022	85
6	Mesenchymal stem cells-derived exosomes are more immunosuppressive than microparticles in inflammatory arthritis	Stella Cosenza	Theranostics	2018	84
7	Oral administration of bovine milk derived extracellular vesicles attenuates arthritis in 2 mouse models	Onno J Arntz	Molecular Nutrition & Food Research	2015	68
8	Exosomal miRNA-486-5p derived from rheumatoid arthritis fibroblast-like synoviocytes induces osteoblast differentiation through the Tob1/BMP/Smad pathway	Jie Chen	Biomaterials Science	2020	64
9	Reactive oxygen species-responsive dendritic cell-derived exosomes for rheumatoid arthritis	Eun Sook Lee	Acta Biomaterialia	2021	58
10	Gender-specific differential expression of exosomal miRNA in synovial fluid of patients with osteoarthritis	Ravindra Kolhe	Scientific Reports	2017	56

### 3.9. Analysis of co-cited references

When multiple research documents cite the same set of references simultaneously, a co-citation relationship forms between the cited documents, reflecting both the associative strength of the research topic and the structure of the knowledge network. Using CiteSpace software, we constructed a co-citation network (Fig. 4B) comprising 2901 nodes and 9653 links, with a network density of 0.0023. Through quantitative citation frequency analysis, we identified the 10 most highly co-cited core references (Table 8), which represent foundational knowledge in this field. The most frequently co-cited reference is a 2020 *Science* review by Kalluri et al,^[24]^ which comprehensively examines the biology, functions, and biomedical applications of EXOs. EXOs participate in diverse physiological and pathological processes – including immune responses, viral infections, pregnancy, cardiovascular diseases, central nervous system disorders, and cancer progression – by delivering proteins, metabolites, and nucleic acids to recipient cells, thereby modulating their biological responses. This cargo delivery can either promote or inhibit disease progression. Key mechanisms include: Immune regulation: Antigen presentation, DNA-induced immune signaling, and miRNA-mediated gene expression. Cancer pathogenesis: Facilitating tumor growth, metastasis, paracrine syndromes, and treatment resistance via miRNA/nucleic acid transfer. Metabolic/cardiovascular roles: Mediating intercellular communication among pancreatic β-cells, adipose tissue, skeletal muscle, and liver through metabolite/miRNA delivery. Neurodegenerative diseases: Influencing misfolded protein aggregation, which may accelerate or mitigate disease. The review also highlights EXOs’ clinical potential. Detectable in all biological fluids, they enable liquid biopsy-based diagnostics. Their complex molecular cargo positions them as ideal platforms for multicomponent diagnostic assays. Moreover, EXOs show promise as therapeutic agents due to their efficient cargo delivery to diseased cells and favorable tolerability in murine models. To elucidate temporal trends, we generated a time zone visualization (Fig. 4C). This analysis revealed significant clustering of highly co-cited literature in 2020, indicating that year’s pivotal role in driving field advancement. Cluster analysis identified 20 distinct thematic clusters (Fig. 4D), each representing a specific research paradigm or hotspot. Timeline visualization further delineated the evolutionary trajectory of these clusters (Fig. 4E), annotating landmark studies with first authors and publication years to map knowledge inheritance. Using an emergence detection algorithm, we identified 20 seminal publications with significant knowledge diffusion effects (Fig. 4F), marking key turning points in the field’s development. Collectively, this multidimensional analysis framework reveals the knowledge structure, temporal distribution patterns, and frontier evolution pathways within the co-cited literature, providing a robust foundation for future research.

**Table 8:** Top 10 co-cited references.

Rank	Title	First author	Journal	Year published	Total co-cited	Centrality
1	The biology, function, and biomedical applications of exosomes	Raghu Kalluri	Science	2020	19	0.00
2	Mesenchymal stem cells-derived exosomes are more immunosuppressive than microparticles in inflammatory arthritis	Stella Cosenza	Theranostics	2018	17	0.00
3	Therapeutic Potential of Mesenchymal Cell-Derived miRNA-150-5p-Expressing Exosomes in Rheumatoid Arthritis Mediated by the Modulation of MMP14 and VEGF	Zhe Chen	Journal of Immunology	2018	16	0.00
4	Exosomes: effectual players in rheumatoid arthritis	Fataneh Tavasolian	Autoimmunity Reviews	2020	12	0.00
5	Anti-inflammatory and immune-regulatory cytokines in rheumatoid arthritis	Zhu Chen	Nature Reviews Rheumatology	2019	9	0.00
6	Rheumatoid arthritis	Josef S Smolen	Nature Reviews Disease Primers	2018	8	0.00
7	Rheumatoid arthritis: pathological mechanisms and modern pharmacologic therapies	Qiang Guo	Bone Research	2018	7	0.00
8	Specificities of secretion and uptake of exosomes and other extracellular vesicles for cell-to-cell communication	Mathilde Mathieu	Nature Cell Biology	2019	7	0.00
9	Gingival mesenchymal stem cell-derived exosomes are immunosuppressive in preventing collagen-induced arthritis	Xiaohong Tian	Journal of Cellular and Molecular Medicine	2022	7	0.00
10	M2-type exosomes nanoparticles for rheumatoid arthritis therapy via macrophage repolarization	Hui Li	Journal of Controlled Release	2022	7	0.00

### 3.10. Exploring the hot spots and evolution of RA and EXOs cross-research based on keywords

**Figure 5.**
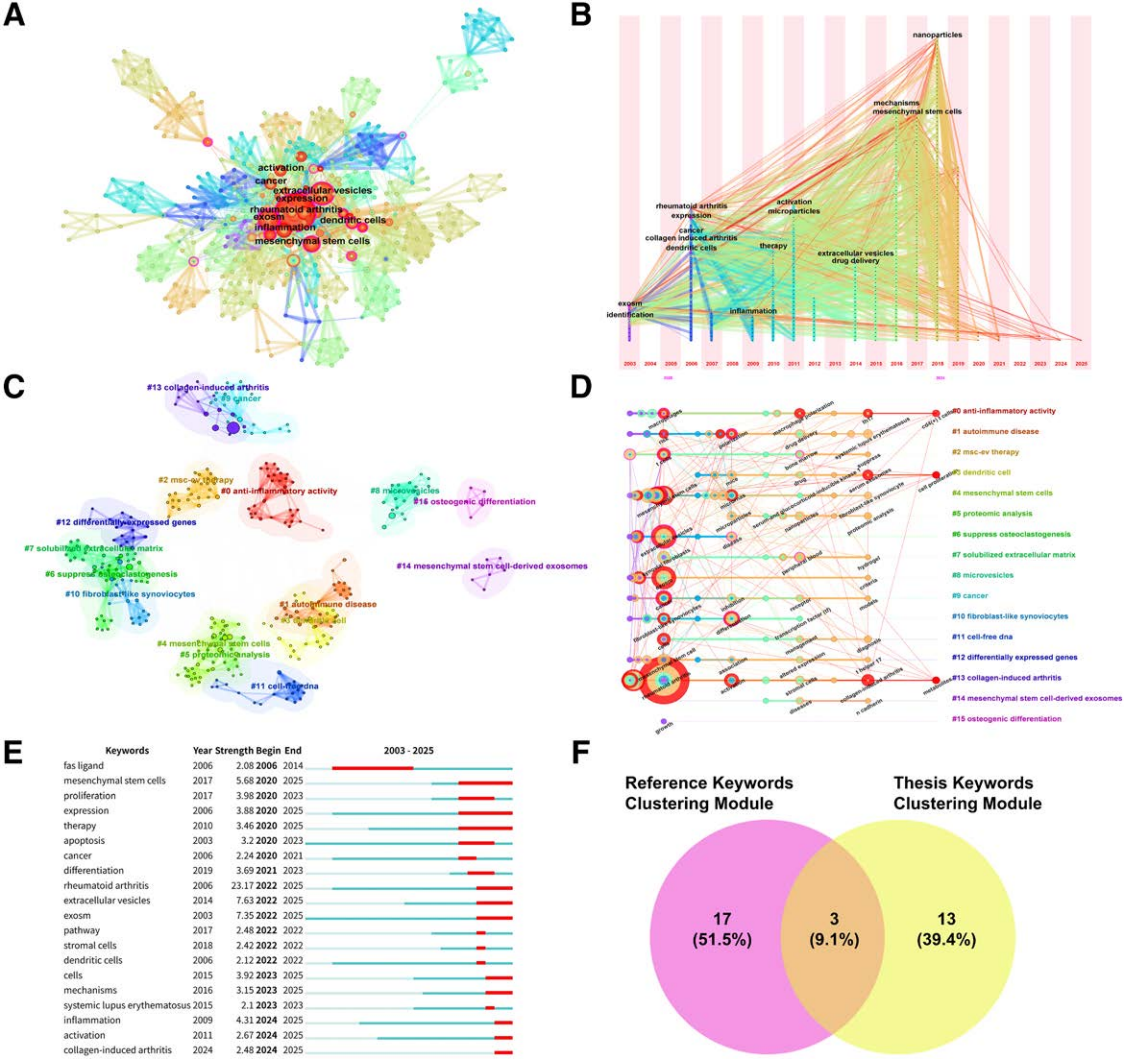
(A) Keyword co-occurrence network. Node size is proportional to frequency of occurrence. (B) Keyword time zone mapping. Node size is proportional to count value. The year “1” is used as the time slice, incrementing from left to right, and keywords whose first occurrence is in the same year are aggregated to the same time zone. (C) Keyword clustering. Different colors represent different clusters. Each node represents a keyword, and the number on the node indicates the cluster to which the keyword belongs. The smaller the number of labels, the more keywords are in that cluster. (D) Keyword clustering timeline. For each cluster, the position of each node represents the time when the keyword appears, and the node size represents the frequency of appearance. (E) Top 20 keywords with the strongest outbreaks. (F) Wayne diagram of keyword clustering of papers versus keyword clustering of co-cited references.

As highly condensed representations of core academic content, keywords provide precise mapping of research topics, scholarly trends, and knowledge structures. The CiteSpace analysis tool generated a keyword co-occurrence network (Fig. 5A), depicting static correlation patterns, and a temporal distribution diagram (Fig. 5B), illustrating dynamic evolutionary trajectories. Table 9 lists the 10 most frequent core keywords, whose distribution reflects current research priorities in the field. Cluster analysis, based on semantic associations, employed intelligent algorithms to automatically categorize keywords with similar meanings. This process formed distinct clusters representing varied research directions, enabling researchers to identify disciplinary substructures and track developmental trends. Per bibliometric standards, a modularity index (*Q*) > 0.3 indicates statistically significant clustering, while a silhouette coefficient (*S*) > 0.7 demonstrates strong cluster homogeneity. As shown in Figure 5C, keyword clustering yielded 16 distinct groups: #0 anti-inflammatory activity, #1 autoimmune disease, #2 MSC-EV therapy, #3 dendritic cell, #4 mesenchymal stem cells, #5 proteomic analysis, #6 suppress osteoclastogenesis, #7 solubilized extracellular matrix, #8 microvesicles, #9 cancer, #10 fibroblast-like synoviocytes, #11 cell-free DNA, #12 differentially expressed genes, #13 collagen-induced arthritis, #14 mesenchymal stem cell-derived EXOs, and #15 osteogenic differentiation. Cluster IDs (#0 to #15) reflect the recency-to-historical evolution of research themes. The values (*Q* = 0.7648 > 0.3; *S* = 0.9265 > 0.7) confirm statistically robust and homogeneous clustering. The largest cluster, #0 (anti-inflammatory activity), likely represents a current research focus. The timeline view (Fig. 5D) further delineates temporal distribution patterns and frequency fluctuations across clusters, enabling vertical comparisons to identify topic emergence, development, and maturation phases. Burst detection identified knowledge leap points by capturing keywords exhibiting significant frequency surges within specific periods. Figure 5E displays the top 20 strongest keyword bursts; their duration and intensity collectively outline the field’s punctuated innovation trajectory. These burst points not only delineate the evolution of research hotspots but also provide critical insights for forecasting emerging directions. Collectively, these complementary visualization approaches establish a comprehensive methodological framework for systematically analyzing knowledge flow dynamics.

**Table 9:** Top 10 frequency and centrality of keywords.

Rank	Keyword	Frequency	Keyword	Centrality
1	rheumatoid arthritis	84	rheumatoid arthritis	0.43
2	expression	18	extracellular vesicles	0.25
3	exosomes	18	mesenchymal stem cells	0.24
4	extracellular vesicles	17	therapy	0.19
5	mesenchymal stem cells	13	differentiation	0.19
6	proliferation	10	expression	0.16
7	inflammation	10	inflammation	0.15
8	therapy	9	classification	0.15
9	differentiation	7	proliferation	0.11
10	apoptosis	7	apoptosis	0.09

## 4. Discussion

### 4.1. General information

In this study, we systematically characterize the literature on RA and EXOs over the past 25 years to map the research landscape and anticipate future scholarly directions. Through bibliometric analysis of 244 qualifying publications, we identified contributions from 1886 authors across 391 institutions in 37 countries/regions. These works were published in 75 journals spanning 31 disciplines and 23 research domains. Key findings include: Temporal trends: Publication output in this interdisciplinary field increased steadily from 2000 to 2025, with total citation frequency rising concurrently, indicating its sustained prominence as a rheumatology research hotspot. National contributions: China leads in both publication volume and academic influence. Seventy percent of the top 10 contributing countries are developed economies, with the United States playing a leading role due to its high output and intermediary centrality. Institutional distribution: research output demonstrates clustering, with the top 10 institutions contributing 38 publications (15.57% of total). Ninety percent of these institutions are based in China. Anhui Medical University produced 11 papers, while Sun Yat-sen University exhibited the highest academic influence. Author impact: among the top 10 authors by average citation frequency, Yao Wang (Southwest Medical University) achieved the highest impact. Researchers should prioritize monitoring developments from high-contributing countries, institutions, and scholars. Implementing systematic literature tracking will help identify paradigm shifts, facilitate publication of original findings in high-impact journals, and advance this field from descriptive studies toward mechanistic analysis and clinical translation.

### 4.2. Hotspots and frontiers

This study employs a multidimensional clustering approach to analyze the literature at the intersection of RA and EXOs. Initially, a keyword co-occurrence network derived from 244 target publications was analyzed using a modularity algorithm, identifying 16 core thematic clusters (Fig. 5C). Subsequently, a literature co-citation network map was constructed, from which hierarchical clustering analysis delineated 20 distinct knowledge domains (Fig. 4D). These 2 sets of clustered data were then comparatively analyzed using matrix overlay. This integration revealed 3 interdisciplinary research areas demonstrating dual significance (Fig. 5F). These areas are characterized by high-density aggregation within the keyword co-occurrence map and occupy central, pivotal positions within the co-citation network structure. Consequently, they represent the current frontier research directions within this interdisciplinary field.

#### 4.2.1. Fibroblast-like synoviocytes

FLS and EXOs drive the pathological process of RA through complex cell-to-cell communication. FLS from RA patients are significantly heterogeneous, and can differentiate into aggressive subpopulations under the action of cytokines, such as TNF-α and IFN-γ. EXOs secreted by such subpopulations carry circular RNAs, such as *circFTO*, and inhibit the expression of SRY-box transcription factor 9 (*SOX9*) in cartilage cells through m6A modification. *SOX9* expression in chondrocytes by m6A modification exacerbates joint damage, and inhibition of exosomal *circFTO* improves arthritis symptoms in mice.^[25]^ Metabolic reprogramming of FLS (e.g., enhanced glycolysis) is closely related to its aggressiveness, and glycolytic enzymes such as hexokinase 2 (*HK2*) are upregulated in RA-FLS, and inhibitors targeting *HK2* selectively inhibit its aggressiveness.^[26]^ EXOs act as mediators of communication between FLS and immune cells and are involved in the remodeling of the RA immune microenvironment. miR-21 carried by RA-FLS EXOs enhances macrophage M1 polarization through the TGF-β signaling axis, forming a positive feedback loop of “FLS-macrophage-fibroblasts.”^[27]^ The CD44 molecule on its surface delivers pro-inflammatory circRNA that activates the NF-κB pathway in T cells and promotes the secretion of IL-6 and IL-17, and lactate dehydrogenase (*LDH*) carried by FLS EXOs enhances the differentiation of Th17 cells through “metabolic hijacking.”^[28]^ Healthy synoviocyte EXOs are enriched in anti-inflammatory *miR-124*, which inhibits the TLR4/NF-κB pathway to reduce inflammation.^[29]^ In addition, EXOs membrane surface integrin αvβ3 activates the FAK/Src signaling pathway in FLS and promotes its invasive behavior. In the development of therapeutic strategies, the University of Science and Technology of China utilized cold air plasma to induce FLS apoptosis and inhibit synovial proliferation.^[30]^ Guoqing Li et al explored the regulation of FLS function by interfering with exosomal circRNA through adeno-associated virus vectors.^[25]^ Mesenchymal stem cell EXOs carrying *miR-320a* inhibit RA-FLS migration and invasion and attenuate symptoms in mice.^[31]^ Nanoenzyme-functionalized neutrophil EXOs target FLS activation, neutralize pro-inflammatory factors and modulate Th17/Treg balance.^[32]^ Cross sectional studies of FLS and EXOs have promoted the development of targeted metabolism, EXOs intervention and localized precision therapy by resolving the mechanisms of heterogeneity regulation, metabolic reprogramming and immune microenvironment remodeling. In the future, the combination of multi-omics technology and engineered EXOs is expected to realize the paradigm shift of RA from inflammation control to tissue repair.

#### 4.2.2. Anti-inflammatory activity

RA, as a chronic autoimmune disease, has a pathology centered on a sustained inflammatory cascade within the synovium. EXOs, due to their natural biocompatibility and targeting properties, are important carriers and therapeutic targets for modulating inflammatory responses and repairing joint damage. By precisely designing the source of EXOs, their surface modifications and the biomolecules they carry, researchers are able to systematically intervene in the inflammatory microenvironment of RA, providing new ideas for precise anti-inflammatory therapy. At the level of molecular mechanisms, EXOs achieve anti-inflammatory effects by targeting and modulating immune cell functions. Mesenchymal stem cell-derived EXOs can inhibit the TLR4/NF-κB pathway and reduce the secretion of TNF-α and IL-6 in synovial fibroblasts by delivering *miR-146a*.^[33]^ Macrophage-derived EXOs, on the other hand, target synovial vascular endothelial cells via surface integrin α4β1 binding, blocking leukocyte infiltration and inhibiting the local inflammatory cascade.^[34]^ This multi-target modulation capability enables EXOs to exhibit anti-inflammatory precision in the RA microenvironment that is superior to that of conventional drugs. Breakthroughs in engineered modification technologies have further enhanced the anti-inflammatory efficacy of EXOs. The charge-allosteric EXOs (MEX+cP) was modified by matrix metalloprotein-sensitive PEG-oligo-lysine to achieve site-specific enrichment and cfDNA clearance at inflamed joint sites, significantly reducing the level of inflammatory factors in the joint cavity in a canine model.^[35]^ Anti-TNF-α antibody-modified EXOs combined with a soluble microneedle delivery system achieves efficient polarization of M1 macrophages to the M2 phenotype in a corneal alkali burn model while increasing drug bioavailability up to 3.2-fold over conventional eye drops.^[36]^ Such functionalized designs not only enhance the targeting of EXOs, but also optimize the anti-inflammatory effect through the spatiotemporal controlled release of signaling molecules. In terms of therapeutic strategy innovation, the synergistic application of EXOs and biomaterials shows great potential. Adipose stem cell EXOs loaded with Icariin system (ADSCs-EXO-ICA) reduced macrophage glycolysis level by inhibiting ERK/HIF-1α/GLUT1 pathway, and decreased IL-1β and TNF-α expression by 67% and 54%, respectively, while promoting cartilage matrix synthesis in a rat model of CIA.^[37]^ M2 macrophage EXOs colocated with IL-10 plasmid and betamethasone nanoparticles achieved a 3.8-fold elevation of IL-10 expression, a 52% reduction in the area of joint destruction, and a reduction in glucocorticoid dosage to one-fifth of the conventional therapy in a mouse model of RA.^[23]^ These studies have confirmed that EXOs not only serve as drug carriers, but also achieve the dual effects of anti-inflammation and tissue repair through the synergistic release of endogenous functional molecules. The in-depth expansion of mechanism studies has provided theoretical support for the anti-inflammatory activity of EXOs. Single-cell sequencing revealed that mesenchymal stem cell-derived EXOs could form an anti-inflammatory microenvironment in the joints by regulating the balance of Th17/Treg cells.^[38]^ EXOs-delivered *miR-22* targets and inhibits the *ATM*/*p53* pathway to reduce synovial cell apoptosis and inflammatory factor release.^[39]^ These findings have elucidated the anti-inflammatory mechanism of EXOs at the epigenetic and cellular metabolic levels, providing a basis for the discovery of novel therapeutic targets. Currently, the field is accelerating towards clinical translation. Dendritic cell-derived EXOs loaded with tretinoin reduced the level of alanine aminotransferase, a marker of hepatotoxicity, by 43% in a model of CIA, while maintaining an equipotent anti-inflammatory effect.^[40]^ With the continuous innovation of engineering technology and in-depth analysis of the mechanism of action, EXOs are expected to drive a fundamental shift in RA treatment from symptom control to immune remodeling in the next 5 to 10 years.

#### 4.2.3. cell-free DNA

In the RA joint microenvironment, cfDNA levels were significantly elevated by the release of mtDNA from apoptotic synoviocytes, neutrophil extracellular traps, and mitochondrial damage.^[41]^ These cfDNAs are both biomarkers of disease activity and drive inflammatory cascade responses through activation of natural immune pathways such as cGAS/STING, TLR9, and others.^[42]^ Down-regulated expression of the DNA degrading enzyme three prime repair exonuclease 1 (*TREX1*) in serum of RA patients leads to cfDNA accumulation and over-activation of the cGAS/STING pathway, exacerbating joint inflammation and bone destruction.^[43]^ EXOs dynamically regulate inflammation by regulating phosphatidylinositol (*PI4P*) and signal-transducing adaptor molecule (*STAM*) proteins in the *TLR4* and cGAS/STING signaling pathways. STING oligomers can be secreted by EXOs and then degraded by lysosomes, allowing for the precise termination of immune signals. The high abundance of mitochondrial DNA fragments in the EXOs of patients with RA activates the cGAS-STING pathway in synovial fibroblasts. STING pathway in synovial fibroblasts, creating an “inflammatory memory” effect.^[44]^ Neutrophil-derived EXOs selectively encapsulate oxidative damage-associated cfDNA, and their N-glycosylation modification pattern can moreover serve as a molecular fingerprint for disease typing, providing a new dimension for targeted intervention. In addition, programmed death-ligand 1 (*PD-L1*) DNA-containing EXOs released by synovial macrophages can induce regulatory T cell dysfunction and build an immunosuppressive microenvironment.^[45]^ Blocking high mobility group box 1 (*HMGB1*), a DNA-binding protein on the surface of EXOs, significantly alleviated joint damage, expanding the traditional “cell-cell” paradigm to a 3-dimensional “EXOs-DNA-receptor” regulatory network.^[46]^ In the field of clinical translation, relevant research results have demonstrated significant potential. Nanopore sequencing-based quantitative analysis of exosomal cfDNA has dramatically increased the sensitivity of early warning of RA compared to traditional antibody detection. MEX+cP specifically binds to cfDNA through surface-modified oligolysine, regulates the immune microenvironment while removing pathogenic DNA, and successfully reverses joint inflammation in animal models.^[35]^ Virus-mimicking exosomal vaccines that activate dendritic cells and induce antigen-specific T-cell responses by delivering fragments of their own DNA open new directions for immunotherapy.^[47]^ Liquid biopsy technology to detect exosomal cfDNA methylation patterns shows promising applications in the early diagnosis and efficacy assessment of RA.^[48]^ These breakthroughs not only deepen the understanding of the molecular mechanisms of RA, but also predict that the EXOs-DNA axis will become a central target for the next generation of immune interventions.

### 4.3. Advantages and limitations

Knowledge graph analysis techniques offer efficient pathways for exploring research dynamics at the intersection of RA and EXOs. This study presents, for the first time, a systematic bibliometric visualization of research trajectories in this interdisciplinary domain; no comparable systematic analysis has been reported to date. However, several limitations warrant acknowledgments. First, data collection relied exclusively on the WoSCC database, potentially limiting the comprehensiveness of included research due to single-source constraints. Second, the analytical tool’s language recognition restricted literature inclusion to English publications, introducing potential selection bias. Finally, the research cycle precluded timely incorporation of new interdisciplinary literature added between compilation and publication, constraining the timeliness of findings. Despite these limitations, the knowledge network constructed herein provides valuable insights for scholars to understand disciplinary evolution and track research frontiers, while also establishing a methodological foundation for subsequent multidimensional, multilingual systematic literature analyses.

## 5. Conclusion

This study conducted a systematic bibliometric analysis of 244 publications at the intersection of RA and EXOs using CiteSpace, spanning the period 2000 to 2025. The analysis revealed research trends, hotspots, and international collaboration patterns within this field. Results indicate a sustained increase in publication volume and significant growth in academic influence. China emerged as the dominant contributor in terms of both literature output (145 publications) and academic centrality (0.57), forming a core research partnership with the United States. High frequency keyword and cluster analyses identified 3 major research frontiers: the mechanism of FLS–EXO interactions, which drive RA progression through metabolic reprogramming and immune microenvironment remodeling; the anti-inflammatory activity of EXOs, underscoring the potential of engineered modifications (e.g., targeted delivery, surface functionalization) for precise regulation of inflammatory cascades; and the role of cfDNA as a key inflammatory mediator. cfDNA exacerbates joint damage by activating innate immune pathways, while EXO-mediated cfDNA clearance strategies offer novel targets for immune intervention. The therapeutic potential of EXOs in RA – particularly in liquid biopsy, drug delivery, and immunomodulation – has been extensively explored. Targeted therapies and tissue repair applications demonstrate considerable promise. This study establishes the first knowledge map of this interdisciplinary field, addresses a critical gap in systematic analysis, and provides a theoretical foundation for future research prioritization, collaboration network optimization, and clinical translation pathway design.

## Author contributions

**Data curation:** Minghui Hou.

**Funding acquisition:** Min Yang.

**Methodology:** Jiayi Ling.

**Software:** Ruilan Liang.

**Writing – original draft:** Zhifeng Zhou.

**Writing – review & editing:** Minglin Ou.
